# Retraction: Antisecretory, Gastroprotective, Antioxidant and Anti-*Helicobcter Pylori* Activity of Zerumbone from *Zingiber Zerumbet* (L.) Smith

**DOI:** 10.1371/journal.pone.0294009

**Published:** 2023-11-10

**Authors:** The PLOS ONE Editors

Following the publication of this article [1], concerns were raised regarding reuse of previously published results in Figs. 2, 3, and 5. Specifically,

- Fig 2A of this study [1] appears similar to Fig 2A of [2, retracted in 3]*, despite being used to represent different experimental conditions.
- Fig 2B of this study [1] appears similar to Fig 2B of [4]*, despite being used to represent different experimental conditions.
- Fig 3B of this study [1] appears similar to Fig 3b of [5]* when rotated, despite being used to represent different experimental conditions.
- Fig 5A of this study [1] appears similar to Figs 10A and 10G of [6, retracted in 7]*, despite being used to represent different experimental conditions.
- Fig 5C of this study [1] appears similar to Fig 5 panel G2 of [8, retracted in 9]*, despite being used to represent different experimental conditions.

The corresponding author stated that the Figs. 2A and 2B of this study [1] and Fig 2A of [2, retracted in 3] and 2B of [4] respectively appear similar because these panels represent the same experimental conditions. However, the methodology reported in these studies suggest that the experimental conditions within the control groups were not identical. Regarding the other overlapping images, the corresponding author stated that incorrect images were inadvertently used during figure preparation. The corresponding author provided replacement panels for Figs. 3B, 5A, and 5C, but these did not resolve the editorial concerns pertaining to the overall data-handling for this study and the reliability of the results presented in this article.

In light of the above concerns, the *PLOS ONE* Editors retract this article.

Some figure panels discussed above appear to report previously published material that are offered under a CC BY license, but the original articles were not attributed in [1]. For these images, the * by the citation, above, marks the oldest publication of the image of which PLOS is aware.

HMAS, NMH, MAA, LMF, and JV agreed with the retraction. HMA, SM, SIA, and MMET either did not respond directly or could not be reached. HMAS, NMH, LMF, and JV apologize for the issues with the published article. MAA stands by the article’s findings.
